# Predicting the suitable habitat distribution of greater amberjack using the MaxEnt model with fishery and remote sensing data in the surrounding waters of Taiwan

**DOI:** 10.1038/s41598-026-60453-6

**Published:** 2026-09-09

**Authors:** Mubarak Mammel, Baker Matovu, Sajna Beegum, Abdul Azeez Pokkathappada, Ming-An Lee, Li-Chi Cheng

**Affiliations:** 1 Department of Environmental Biology and Fisheries Science, National Taiwan Ocean University, No.2, Beining Rd., Zhongzheng Dist, Keelung City, 20224 Taiwan; 2 Future Earth Coasts (FEC) Fellow, Kampala, Uganda; 3 Department of Sustainable Development, Blue Economy, and Ocean Futures, Africa International Policy Research Center, Nairobi, Kenya; 4 Government College of Education, Affiliated with Pondicherry University, Kadmat Island, Lakshadweep, India; 5 ICAR-Central Marine Fisheries Research Institute, Kochi, 682018 Kerala India; 6 Doctoral Degree Program in Ocean Resource and Environmental Changes, National Taiwan Ocean University, No.2, Beining Rd., Zhongzheng Dist, Keelung City, 20224 Taiwan; 7 Center of Excellence for Oceans, National Taiwan Ocean University, No.2, Beining Rd., Zhongzheng Dist, Keelung City, 20224 Taiwan; 8 Center for Humanities and Social Sciences in Governance, National Taiwan Ocean University, No.2, Beining Rd, Zhongzheng Dist, Keelung City, 20224 Taiwan; 9 Coastal and Offshore Fishery Research Center, Fisheries Research Institute, Ministry of Agriculture, No. 6 Yugang N. 3rd Rd., Qianzhen Dist, Kaohsiung City, 806043 Taiwan

**Keywords:** Habitat suitability index, Maximum entropy model, Satellite data, Oceanographic variable, Habitat selection, Ecology, Ecology, Environmental sciences, Ocean sciences

## Abstract

The increasing availability of satellite-derived remotely sensed oceanographic data offers significant potential for evaluating changes in habitat suitability driven by oceanographic phenomena and for informing scientific management strategies, particularly when coupled with maximum entropy (MaxEnt) methods. Oceanographic variables, including sea surface temperature (SST), sea surface salinity (SSS), sea surface height (SSH), sea surface chlorophyll-a concentration (CHL), mixed layer depth (MLD), and eddy kinetic energy (EKE), alongside fishery data from Taiwanese fishing vessels, were collected for the period 2014–2019. The MaxEnt model is a widely employed method for predicting species’ geographical distributions by analyzing species occurrence data in relation to environmental variables. Our results indicated that the annual response curves of habitat suitability were significantly influenced by environmental factors. Specifically, optimal environmental conditions for high habitat suitability were identified as SST > 24 °C, SSS < 35 PSU, SSH between 0.41 and 0.64 m, CHL > 0.22 mg/m^3^, MLD is between 10 and 17 m, and EKE < 0.007 m^2^/s^2^. The MaxEnt models exhibited strong predictive performance across all seasons and overall, as evidenced by receiver operating characteristic curve (AUC) values exceeding 0.8 and true skill statistics (TSS) values above 0.7, thereby confirming their accuracy in predicting greater amberjack presence, a reference for future conservation and management priorities.

**Supplementary Information:**

The online version contains supplementary material available at https://doi.org/10.1038/s41598-026-60453-6.

## Introduction

Species distribution modeling is fundamentally based on the ecological niche concept, which defines the environmental parameters that support positive population growth for a given species^1^. This methodological approach involves quantifying the relationships between a species’ geographical distribution and various environmental variables, thereby enabling accurate predictions of potential habitats^2^. A thorough understanding of these environmental associations is critical for effective conservation and management efforts, particularly for marine species that frequently exhibit distinct habitat preferences^3,4^. For example, fish species commonly select habitats optimized for activities such as foraging, reproduction, and migration, rendering oceanographic parameters indispensable for predicting fishing grounds^5^. Predictive habitat modeling, employing algorithms like maximum entropy, is becoming an increasingly vital tool for marine ecologists and conservation scientists to estimate species distribution patterns and formulate robust conservation strategies^4,6^. These models offer significant advantages, especially when data are limited or consist solely of presence records, a common challenge in marine fisheries research^7^. Moreover, they facilitate the identification of key environmental drivers influencing species distribution, which is essential for understanding ecological responses to climate change and anthropogenic pressures. Advanced habitat modeling techniques, such as those that integrate satellite-derived oceanographic data and species distribution models, have substantially improved the capacity to analyze and predict species’ habitat preferences and spatial distributions^1,8^. Specifically, maximum entropy models have emerged as robust instruments for predicting potential suitable habitats for a variety of marine species, including pelagic fishes, by effectively utilizing presence-only data and machine learning techniques^7,9^. This methodology has demonstrated particular efficacy in situations where comprehensive absence data are unavailable, relying instead on the principle of maximizing entropy to infer the most uniform possible distribution within specified environmental constraints^10^.

Greater amberjack, a significant species for commercial fisheries, recreational angling, and aquaculture, is classified as a reef-associated carangid. Its distribution spans tropical and temperate marine environments across the Atlantic, Pacific, and Indian Oceans^11,12^. Specifically, within the Carangid family, the greater amberjack constitutes the largest pelagic contingent and is characterized as an opportunistic predator, commonly observed in proximity to reef structures and pelagic floating objects^3^. Prior research on pelagic species, such as albacore tuna, consistently emphasizes the critical role of oceanographic parameters in determining species distribution patterns and habitat suitability^13^. These intricate dynamics highlight the imperative of integrating environmental variables into predictive models to accurately forecast the spatiotemporal distribution of species such as the greater amberjack^11^. The oceanographic dynamics around Taiwan are predominantly shaped by the Kuroshio Current, the South China Sea Current, and the China Coastal Current, which collectively exert a significant influence on nutrient dispersion and thermal stratification, pivotal for supporting marine ecosystems^14^. These current systems foster a highly dynamic environment conducive to a diverse array of marine biota, encompassing commercially valuable species such as skipjack tuna and various grouper species^15^. The convergence of these current systems, particularly within areas like the Taiwan Strait, instigates upwelling phenomena, thereby augmenting primary productivity and delineating critical fishing areas^16^. Specifically, the western Taiwan region experiences seasonal modulations in these current patterns, resulting in varied oceanographic conditions which subsequently influence prey distribution and the foraging ecology of species such as the greater amberjack^3,12^. For instance, the greater amberjack’s distribution in Taiwanese waters exhibits a strong correlation with distinct ranges of sea surface temperature (SST), sea surface salinity (SSS) and sea surface chlorophyll-a concentration (CHL), underscoring the profound influence of these environmental parameters on its habitat preferences^17^. The Kuroshio Current, flowing into the East China Sea east of Taiwan, is also acknowledged for sustaining substantial populations of foraging species, even amidst characteristically oligotrophic conditions, thereby emphasizing its indirect, yet significant, contribution to the maintenance of diverse marine ecosystems^18^. This complex interaction of oceanographic processes directly modulates the availability of prey resources, which profoundly impacts the trophic dynamics and spatial distribution of piscivorous predators like the greater amberjack^8,19^.

Maximum Entropy (MaxEnt) modeling is a machine learning method that predicts the geographical distribution of a species by analyzing occurrence data and environmental variables^7,9^. This approach is particularly effective for species with limited occurrence records, such as the greater amberjack in Taiwanese waters, where comprehensive abundance data are often scarce^3^. By leveraging environmental parameters such as SST, CHL, and ocean currents, MaxEnt models can delineate potential habitats, providing crucial insights for conservation and fisheries management. This methodology allows for the identification of optimal environmental conditions and spatial patterns linked to the distribution of species, as demonstrated by studies on skipjack tuna and other marine organisms^1^. Furthermore, the integration of remotely sensed data with MaxEnt models has proven instrumental in enhancing the accuracy of species distribution predictions, especially for highly mobile pelagic species like the greater amberjack^11,20^. These models consider how environmental variables like SST, CHL, and SSS influence species distribution, often revealing specific optimal ranges for different life stages or activities^5^. This approach has been successfully applied to predict the distribution of a wide range of marine organisms, from reef-associated fish to highly migratory pelagic species, offering critical insights into their ecological niches and responses to environmental changes^4,10^.

This study aims to develop a habitat suitability index (HSI) model to predict the fishery potential and future habitat shifts for greater amberjack in the waters surrounding Taiwan. Specifically, it will utilize a MaxEnt model to predict the species’ habitat suitability, analyze key oceanographic environmental characteristics and their associated Suitability Indices (SI), and assess both the seasonal and overall habitat distribution of greater amberjack in the adjacent coastal Taiwanese waters and the broader surrounding region.

## Materials and methods

### Study area and target species

**Fig. 1:**
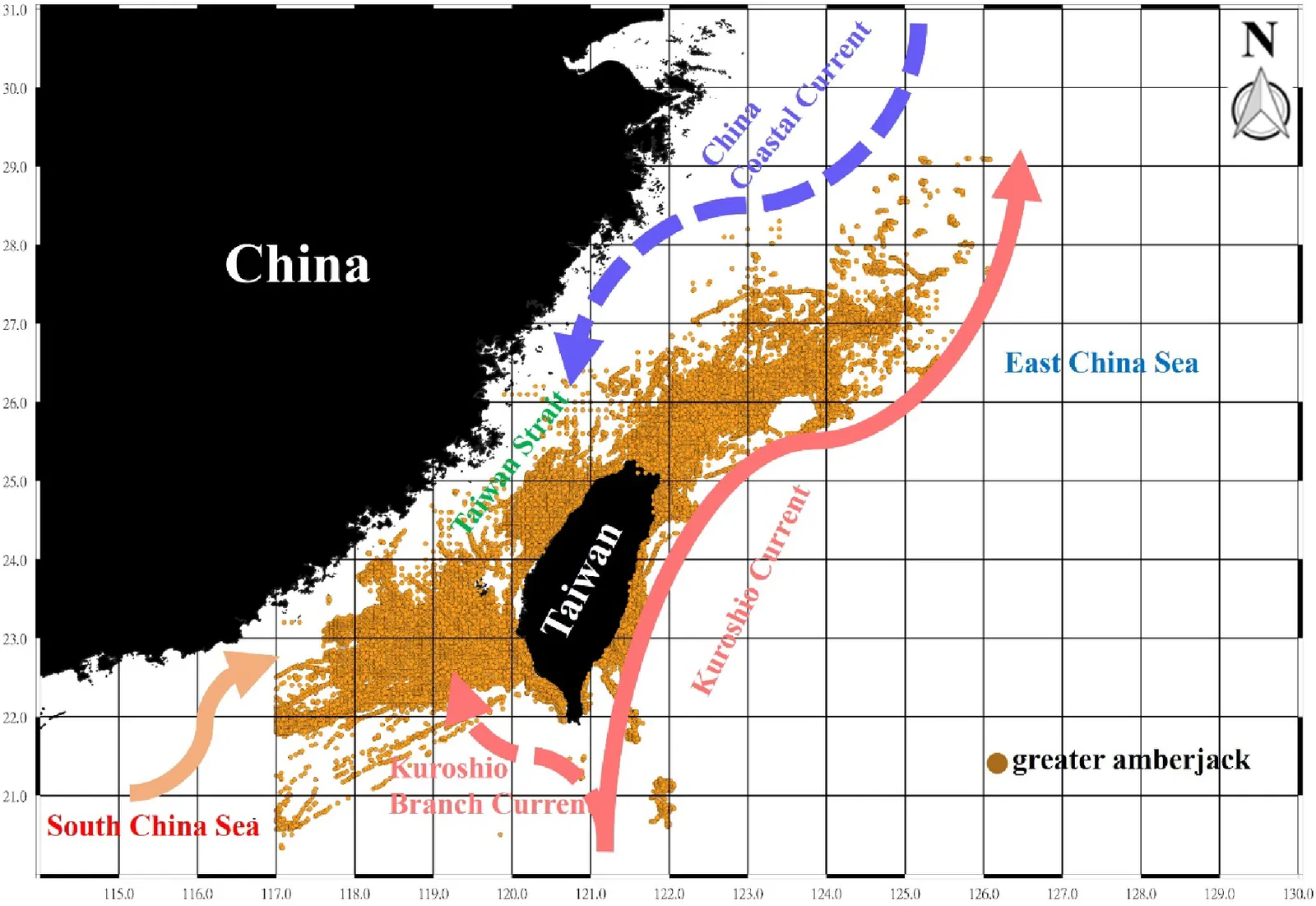
Distribution of greater amberjack catch points shows in the small brown dots during 2014–2019. The arrows represent the current patters surrounding the waters of Taiwan. Map created by the authors using Quantum GIS (QGIS, version 3.6; https://www.qgis.org).

This study focused on the oceanographic region surrounding Taiwan, encompassing the area between 20°N–30°N and 115°E–128°E (Fig. 1). Spatial mapping and visualization of the study area and greater amberjack fishery distribution were performed using Quantum GIS (QGIS, version 3.6). This region is characterized by ecologically crucial marine habitats that support diverse pelagic species and dynamic oceanographic conditions. It is particularly relevant for understanding habitat suitability in relation to oceanographic environmental variability and serves as a central hub for fisheries activity in the northwestern Pacific.

Data concerning greater amberjack were collected from Taiwanese fishing vessels equipped with rod-and-reel angling gear, operating in the waters surrounding Taiwan. These encompassed areas within the South China Sea south of Taiwan and extending into the East China Sea north of Taiwan, spanning the period from 2014 to 2019. The spatial distribution patterns of greater amberjack within Taiwan’s surrounding waters are illustrated in Fig. 1. Fishing data, recorded within 0.1° spatial grids, comprised daily fishing locations (latitude and longitude), fishing dates, operating hours, total catch by weight, and target species. From the comprehensive Voyage Data Recorder (VDR) datasets, only information related to angling gear, identified as the predominant method for targeting greater amberjack, was extracted for this study. Fishing effort was quantified as work hours (whr), and catch was normalized by the proportion of effort at each location relative to total effort (Eq. 1). Catch rates were subsequently derived as an effort-weighted index in units of kg/hr (Eq. 2), following the approach established in our previously published studies on greater amberjack in Taiwanese waters^3,11^. Seasonal fishery data for greater amberjack were compiled from the VDRs of Taiwanese fishing vessels operating in the waters surrounding Taiwan between 2014 and 2019 (Fig. S1). Given the extensive information contained within VDRs, raw data underwent a rigorous filtering and cleaning process, involving appropriate calculations, to prepare them for subsequent use in the fishing detection model.

   

1 $$\:{Catch}_{modified}=\left(\frac{Work\:hour}{Total\:work\:hour}\right)\times\:Weight\:\left(kg\right)$$

  

2 $$\:Catch\:Rate\:\left(\raisebox{1ex}{$kg$}\!\left/\:\!\raisebox{-1ex}{$hr$}\right.\right)=\frac{{Catch}_{modified}\left(kg\right)}{Fishing\:effort\:\left(hr\right)}$$

### Oceanographic environmental variables

Previous investigations have recognized SST, SSS, sea surface height (SSH), CHL, mixed layer depth (MLD), and eddy kinetic energy (EKE) as crucial variables influencing the spatial distribution of greater amberjack^3,11^. The spatiotemporal distribution of greater amberjack is intrinsically linked to subsurface environmental conditions, which can also consequently affect the distribution of target species and the operational patterns of surface fishing vessels. Monthly oceanographic environmental data were acquired from the Copernicus Marine Environment Monitoring Service (CMEMS) (https://marine.copernicus.eu). SST data, with a spatial resolution of 1/12°, were obtained from CMEMS. SSH serves as an indicator for ocean current dynamics, eddy formation, fronts, and convergence zones. Monthly CHL data were derived from the National Aeronautics and Space Administration Aqua (NASA) satellite, utilizing observations from the Moderate Resolution Imaging Spectroradiometer sensor for global ocean CHL detection (https://oceancolor.gsfc.nasa.gov). The CHL dataset provided a monthly temporal resolution and a spatial resolution of 4.6 km for its level 3 standard map imagery. EKE was computed based on zonal (U) and meridional (V) current components using the formula (EKE = 0.5 (U^2^ + V^2^)). Comprehensive details regarding each variable are presented in Table 1. To align with the spatial resolution of the fishery data, these remotely sensed environmental variables were resampled and processed into monthly averages on a 0.1° spatial grid using the MATLAB software (version R2019a). To harmonize the temporal resolution between datasets, daily fishery catch records were aggregated into monthly totals within each 0.1° spatial grid cell and subsequently matched with the corresponding monthly environmental layers. This temporal matching ensured consistency between the fishery-dependent presence data and the environmental predictors used in the MaxEnt model. These oceanographic environmental variables were derived from the acquired remote sensing environmental data. Given the importance of seawater temperature and oceanic fronts for the distribution of large pelagic species, the seasonal spatial distribution patterns of SST were analyzed and are presented in the supplementary section (Fig. S2). The oceanic front detection algorithm, developed by^21^ and based on SST satellite imagery, was employed to calculate the magnitude of the SST front gradient (Fig. S3).

**Table 1:** Remotely sensed environmental variables and descriptions applied in the MaxEnt model.

Environmental variables	Abbreviation	Units	Data source	Temporal resolution	Spatial resolution
Sea Surface Temperature	SST	°C	http://marine.copernicus.eu	Monthly	1/12°
Sea Surface Salinity	SSS	PSU	http://marine.copernicus.eu	Monthly	1/12°
Sea Surface Height	SSH	m	http://marine.copernicus.eu	Monthly	1/12°
Sea Surface Chlorophyll-a Concentration	CHL	mg/m^3^	oceancolor.gsfc.nasa.gov	Monthly	4.6 km × 4.6 km
Mixed Layer Depth	MLD	m	http://marine.copernicus.eu	Monthly	1/12°
Eddy Kinetic Energy	EKE	m^2^/s^2^	http://marine.copernicus.eu	Monthly	1/12°

Multicollinearity, a potential issue among environmental variables^22^ necessitates careful consideration. High correlations between these variables can lead to inaccurate interpretations of their individual percent contributions and render marginal response curves misleading^23^. To mitigate this, the variance inflation factor (VIF) was computed to reduce multicollinearity among the environmental variables used in species distribution model analysis. In addition to VIF analysis, a pairwise Pearson correlation matrix among all environmental predictor variables was computed to further assess inter-variable relationships and potential redundancy among predictors (Fig. S4). Elevated VIF values indicate a higher likelihood of collinearity among independent variables. Consistent with established practices, collinearity is typically addressed by removing variables from the analysis when their VIF values surpass predetermined thresholds^24^. In this study, variable selection was performed using a VIF threshold of 5, meaning only variables with VIF values below this threshold were incorporated into the MaxEnt analysis. A distinct MaxEnt model was subsequently developed utilizing only these selected variables.

### MaxEnt model construction

The MaxEnt model, a machine learning algorithm, employs maximum entropy theory to estimate the influence of environmental factors on species distribution^23^. Developed for presence-only habitat suitability modeling, it demonstrates high predictive accuracy^25^. Analyses in this study were conducted using MaxEnt software (version 3.4.1), applying its default settings. These default parameters are generally recommended for non-expert users due to their automated complexity control, and previous studies have validated their efficacy across diverse species datasets and groups^25,26^. Environmental parameters were input into MaxEnt using the samples-with-data format, rather than raster files. This format is preferred for its applicability to numerous environmental grids and its suitability for long-term records involving various environmental conditions^27^. To ascertain the effects of fishery data types, MaxEnt models were constructed using VDR catch records, specifically focusing on catch type and fishing effort data from Taiwanese fishing vessels. Only grid cells with catch rate > 0 were retained as presence records in the MaxEnt model. Zero-catch records where fishing effort was recorded but no catch was made were excluded from the presence dataset. Grid cells with no recorded fishing effort were designated as pseudoabsence candidates, consistent with the assumption that fishing activity at a location reflects a deliberate decision by the fishing master based on perceived fish availability. The permutation and percentage contribution of environmental factors to the MaxEnt models were determined via the standard jack-knifing function^28^.

For a 0.1° spatial grid, the habitat suitability scores for greater amberjack were determined using the MaxEnt model. Prior to model fitting, fishery and environmental data were matched using a 0.1° spatial grid to ensure spatial consistency across datasets. This grid-based aggregation inherently reduces spatial clustering by consolidating multiple fishing records within the same grid cell into a single presence point, thereby partially mitigating spatial autocorrelation in the occurrence data. Spatial autocorrelation in occurrence data is a recognized issue in species distribution modelling that can inflate model performance metrics and reduce the effective independence of training samples. The use of a 0.1° spatial grid in this study serves as a form of spatial rarefaction, ensuring that presence records are spatially distinct at the resolution of the environmental predictors. This correlative modeling approach relies on the occurrence data of the species within the study area^25^. Fishing catch data for greater amberjack from 2014 to 2018 were employed for model training, while data from 2019 were reserved for predictive analysis. Separate MaxEnt models were developed for each meteorological season: spring (March–May), summer (June–August), autumn (September–November), and winter (December–February), in addition to one overall annual model. This seasonal stratification explicitly captures temporal variability in habitat use, as reflected in the seasonal/annual response curves and variable contribution plots. Within the MaxEnt model, presence probability values range from 0 to 1, with elevated values signifying more favorable environmental conditions for fish distribution; specifically, probabilities exceeding 0.6 were identified as indicative of suitable habitats^3,29^.

All models were constructed employing the default settings for the regularization parameter. A total of 10,000 background points were utilized for the 0.1° spatial resolutions, with a maximum of 500 iterations and automatic feature selection. Specifically, the default MaxEnt configuration included a regularization multiplier of 1.0 and automatic selection of feature classes (linear, quadratic, hinge, product, and threshold features). The regularization multiplier helps control model complexity and reduce the risk of overfitting^25^. These background points, selected by default from the geographical space, were treated as pseudoabsences. Fishery-dependent data from Taiwanese commercial fisheries served as presence data, as empirical absence data for greater amberjack were unavailable. Pseudoabsence data, crucial for evaluating model performance, were generated from the entire study area using a one-step strategy to enhance accuracy^30^. While the generation of pseudoabsence data is a standard practice for MaxEnt models, it presents limitations, such as the potential inaccuracy of randomly selected pseudoabsence points in reflecting actual absence. Specifically, for VDR data, areas devoid of catch records were designated as pseudoabsence points. We acknowledge, however, that the absence of fishing effort does not necessarily indicate the absence of the species, as vessels may avoid certain areas due to operational constraints such as distance from port, weather conditions, fuel costs, regulatory restrictions, or individual skipper strategy. This introduces a potential systematic bias toward fishing effort patterns rather than true ecological unsuitability. Nevertheless, the use of 10,000 random background points drawn from the entire study area partially mitigates this issue by sampling broadly across the full environmental space, rather than being restricted to fished areas alone. Furthermore, the well-documented behavioral ecology of greater amberjack in Taiwanese waters particularly their strong association with reef structures and oceanographic frontal zones provides ecological justification for linking fishing effort locations with likely species presence, as experienced fishing masters in this artisanal rod-and-reel angling fishery actively seek aggregations based on accumulated localized ecological knowledge.

Response curves were generated, and a jackknife test was employed to assess the relative importance of variables within the model. These curves were subsequently utilized to illustrate the spatial distribution probability of greater amberjack fishing operations as a function of environmental variables. For each season and for the models overall, the presence data were randomly partitioned into 70% for training and 30% for validation. The training dataset facilitated model fitting, while the validation dataset was employed to evaluate model performance. This random splitting of observations into training and testing datasets is a standard k-fold cross-validation technique, which enhances the robustness and reliability of model predictions^31^.

### Model performance assessment

Fishery data and associated environmental variables were subsequently utilized to validate the chosen habitat model. Environmental data, both seasonal and overall, served as inputs for predicting the spatial distribution of greater amberjack habitat. The resulting seasonal and overall spatial patterns of these habitats were assessed by averaging the spatial predictions across multiple seasons. The goodness of fit for the MaxEnt models was evaluated using the area under the curve (AUC) of the receiver operating characteristic (ROC) curve of the validation dataset^32^. Model performance was assessed by comparing predictions in spatial grids where the species was observed against those where it was absent or unobserved, thereby enhancing specificity^33^. It is important to note that the AUC generated by MaxEnt compares presences against background data, which may not represent true absences^23^. Consequently, the reported AUC is referred to as a presence-only AUC to maintain clarity regarding this distinction^34^. AUC values range from 0 to 1, where 0.5 signifies performance no better than random chance; scores between 0.7 and 0.9 are generally considered useful for forecasting^32^. In this study, a high AUC value, specifically exceeding 0.8, was interpreted as an indicator of excellent model performance^32^; however, it is important to note that AUC values derived from MaxEnt reflect presence-background discrimination rather than true presence-absence performance, and may be inflated depending on the spatial extent and composition of the background data. Accordingly, AUC values should be interpreted with caution and not directly compared to those from presence-absence models. Future studies should consider complementary evaluation metrics such as the continuous Boyce index, which is less sensitive to background selection and more appropriate for evaluating presence-only models. The validated best-fitting monthly models were employed to calculate HSI for the periods 2014–2018 and 2019. Spatial maps were subsequently generated by overlaying these HSIs with actual fishing operation locations to assess the models’ predictive accuracy. Nevertheless, caution is advised when interpreting AUC values, as a perfect model might sometimes yield low AUC, and a high AUC could potentially be influenced by the proportion of absent background data^34^. Greater amberjack fishery data were partitioned, with 2014–2018 used for model generation and 2019 reserved for validating the model’s predictive capability. The determination of appropriate thresholds necessitates considering the relative importance of both omission and commission errors. True skill statistics (TSS)^35^ is a widely recognized metric for assessing these error types^36^. TSS values range from 0 to 1, where higher values denote superior model performance^35^. In the present study, both AUC and TSS values, derived from test and training sets, served as indicators for evaluating the overall model performance.

## Results

### MaxEnt model performance assessment and selection criteria

VIF analysis indicated that multicollinearity was not a significant concern among the oceanographic variables, as all VIF values remained below the accepted threshold (Fig. S5). The MaxEnt models demonstrated robust performance, with AUC values exceeding 0.8 for both training and testing datasets across all seasons and overall (Table 2). It should be noted, however, that these AUC values represent presence-background discrimination and may be subject to inflation given the spatial extent of the study area and the composition of the background points. Therefore, these values should be interpreted as indicative of model discriminatory ability rather than absolute predictive accuracy. This high performance suggests that the selected environmental variables play significant roles in determining habitat suitability for greater amberjack. The habitat suitability simulations exhibited strong discriminatory ability, confirming the model’s adequacy for investigating the distribution of suitable greater amberjack habitats in Taiwanese waters. Furthermore, TSS values for both seasonal and overall models consistently exceeded 0.7, further reinforcing the indication of strong model performance (Table 2).

**Table 2:** Results of a MaxEnt model performance of the AUC and TSS values for each season and overall.

	Spring	Summer	Autumn	Winter	Overall
Training AUC	0.944	0.996	0.994	0.855	0.922
Test AUC	0.936	0.996	0.993	0.835	0.914
TSS	0.726	0.991	0.957	0.543	0.702

### Environmental variable contributions and their relative importance in the MaxEnt model

**Fig. 2:**
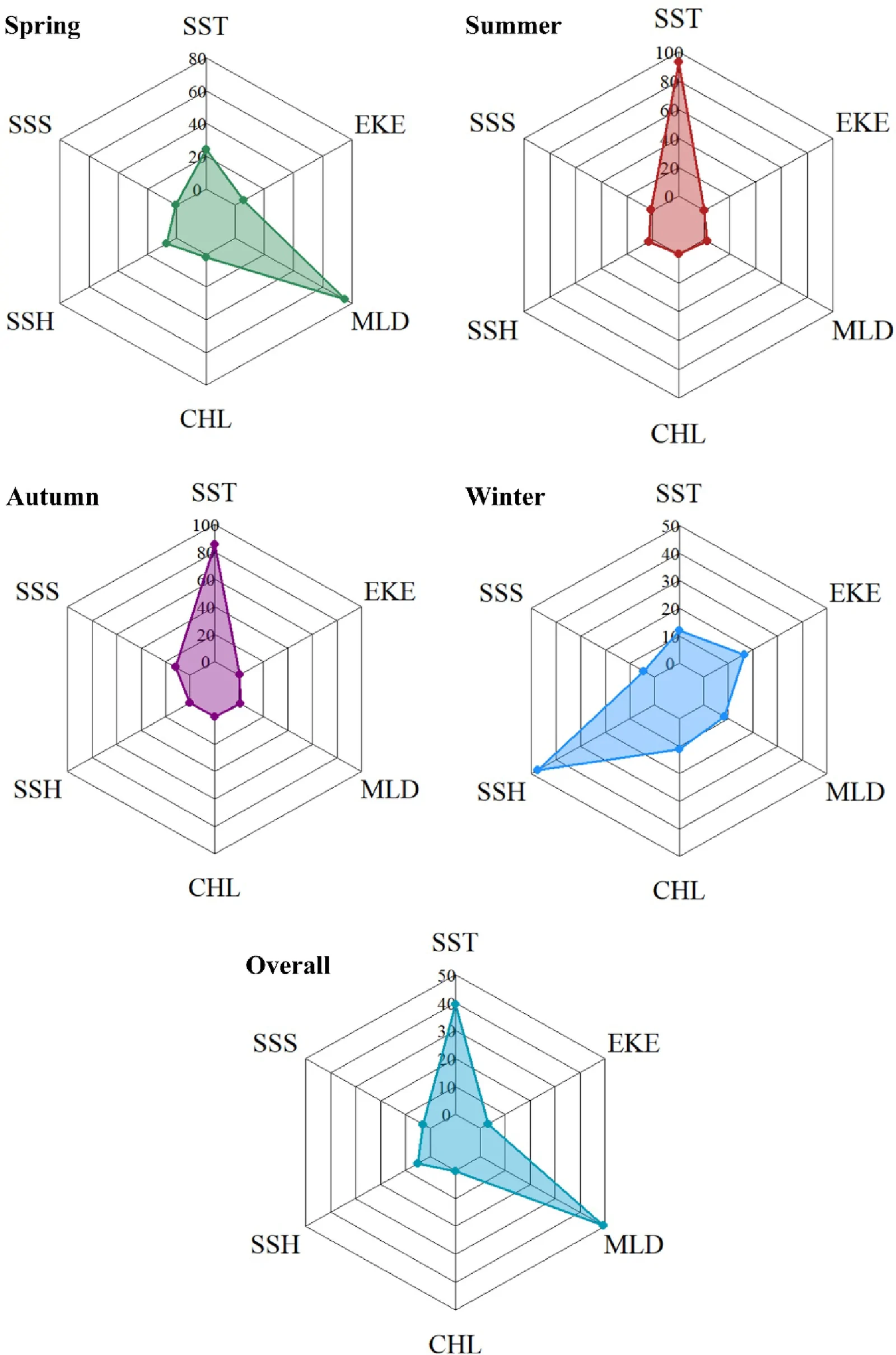
The relative contribution-based correlations of each environmental variable by seasonal MaxEnt models.

The percentage contributions of environmental factors within the MaxEnt model, evaluated both seasonally and overall, as illustrated in (Fig. 2), indicated a discernible seasonal trend where the correlation of a single factor was predominantly significant. Specifically, during summer and autumn, SST exhibited the highest contribution, consistently exceeding 80%. In contrast, MLD showed the strongest correlation in spring, with contributions above 60%, while SSH was most prominent in winter, contributing over 40%. Conversely, the overall model indicated that MLD and SST were the primary contributors to the distribution of habitat suitability for greater amberjack. The relative importance of environmental factors, as determined by various pelagic habitat models, demonstrated considerable variability. Furthermore, jackknife analysis of the MaxEnt model results identified critical environmental variables for each season (Fig. S6) and for the overall dataset pertaining to greater amberjack (Fig. S7).

### Assessment of oceanographic habitat suitability for greater amberjack

**Fig. 3:**
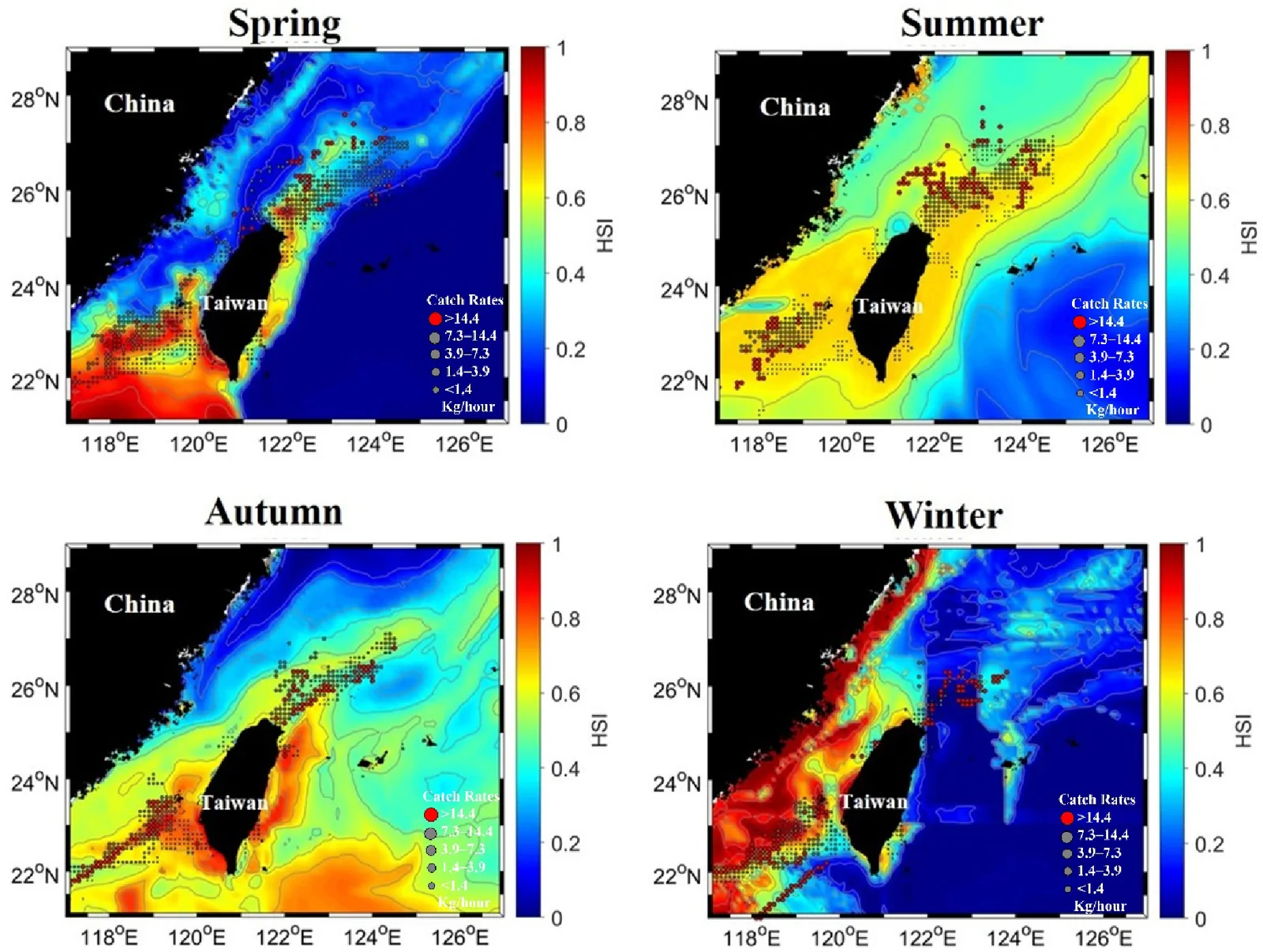
The seasonal distribution of habitat suitability derived from MaxEnt model for greater amberjack. Map generated using MATLAB software (version R2019a; https://www.mathworks.com).

**Fig. 4:**
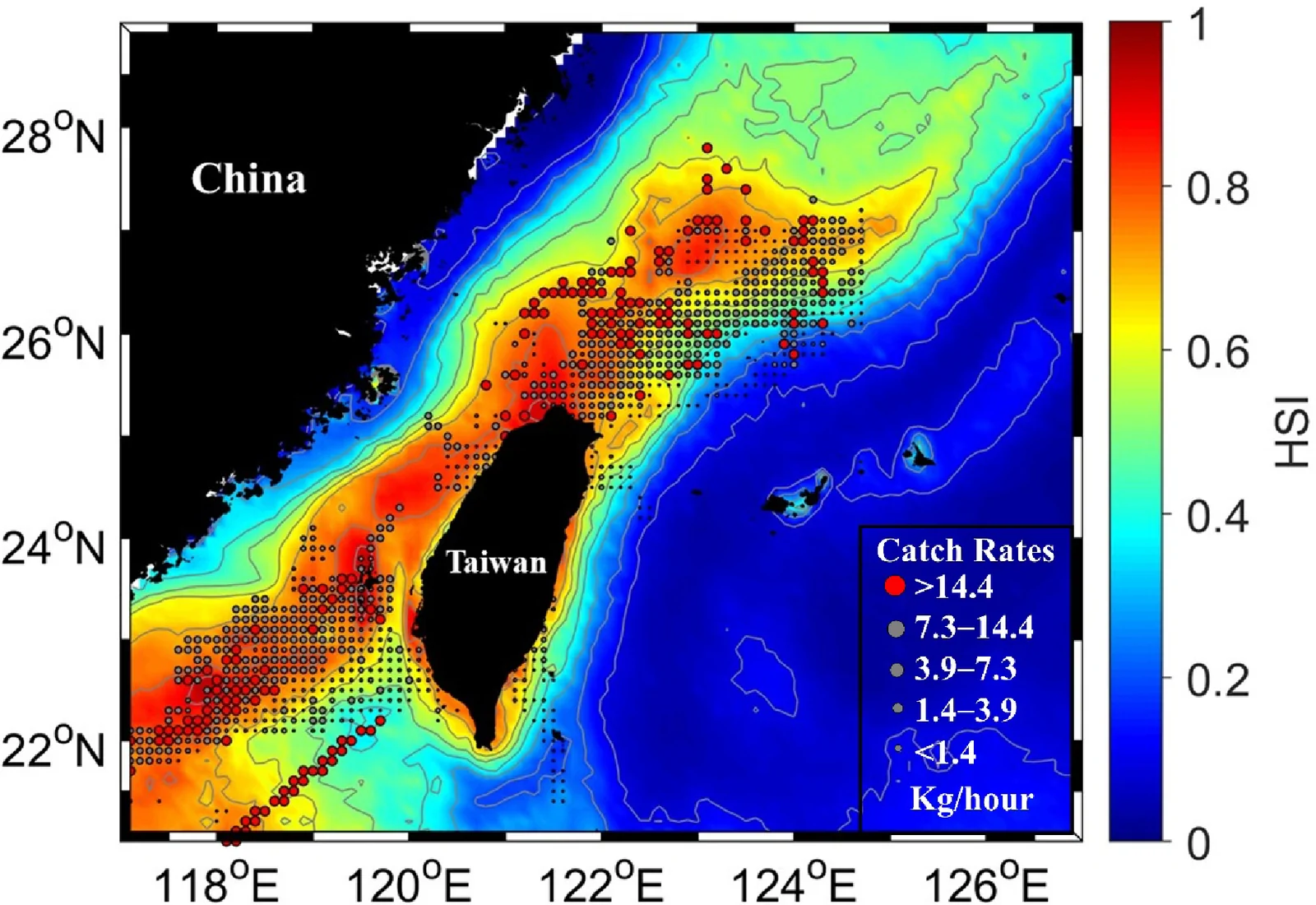
The overall distribution of habitat suitability derived from MaxEnt model for greater amberjack. Map generated using MATLAB software (version R2019a; https://www.mathworks.com).

Figure 3 illustrates the seasonal predictions of the HSI for greater amberjack, juxtaposed with the corresponding seasonal average catch rates observed in the waters surrounding Taiwan. These catch rates were crucial for accurately predicting the primary fishing grounds with high spatial resolution. Consequently, the predicted distribution revealed a notable concentration in the southwestern and as well as some northern region of Taiwan during spring. In summer, elevated HSI predictions were observed in the southwestern area and across the northwestern region, extending into the waters of the East China Sea. The habitat range demonstrated a gradual contraction, reaching its broadest extent in autumn, followed by a subsequent expansion across the northwestern and northeastern waters of Taiwan. Nevertheless, the most suitable habitat was predominantly projected along the western coast of Taiwan and within the coastal waters of mainland China in winter. Critically, the overall HSI predictions exhibited a precise concordance with observed catch rates, wherein higher HSI values directly aligned with increased catch rates (Fig. 4). This agreement validates the reliability of the MaxEnt model, affirming its robust consistency with remotely sensed environmental variables and the recorded catch rates of greater amberjack fisheries in Taiwanese waters.

### Environmental response curves and habitat suitability delineation

**Fig. 5:**
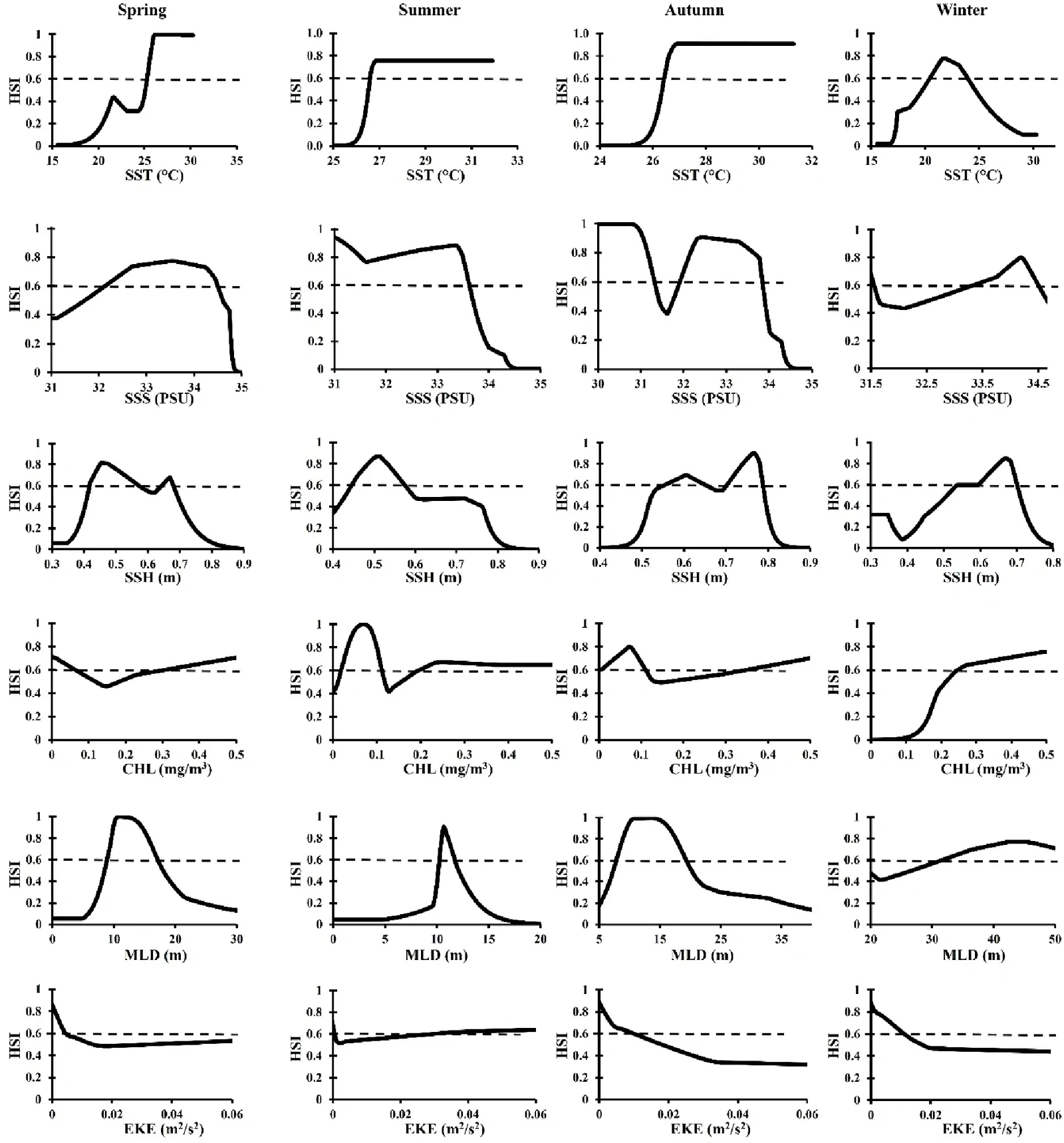
Response curves of environmental variables to habitat suitability derived from the MaxEnt model by season. Horizontal dashed lines indicate suitable environmental variable ranges for greater amberjack.

**Fig. 6:**
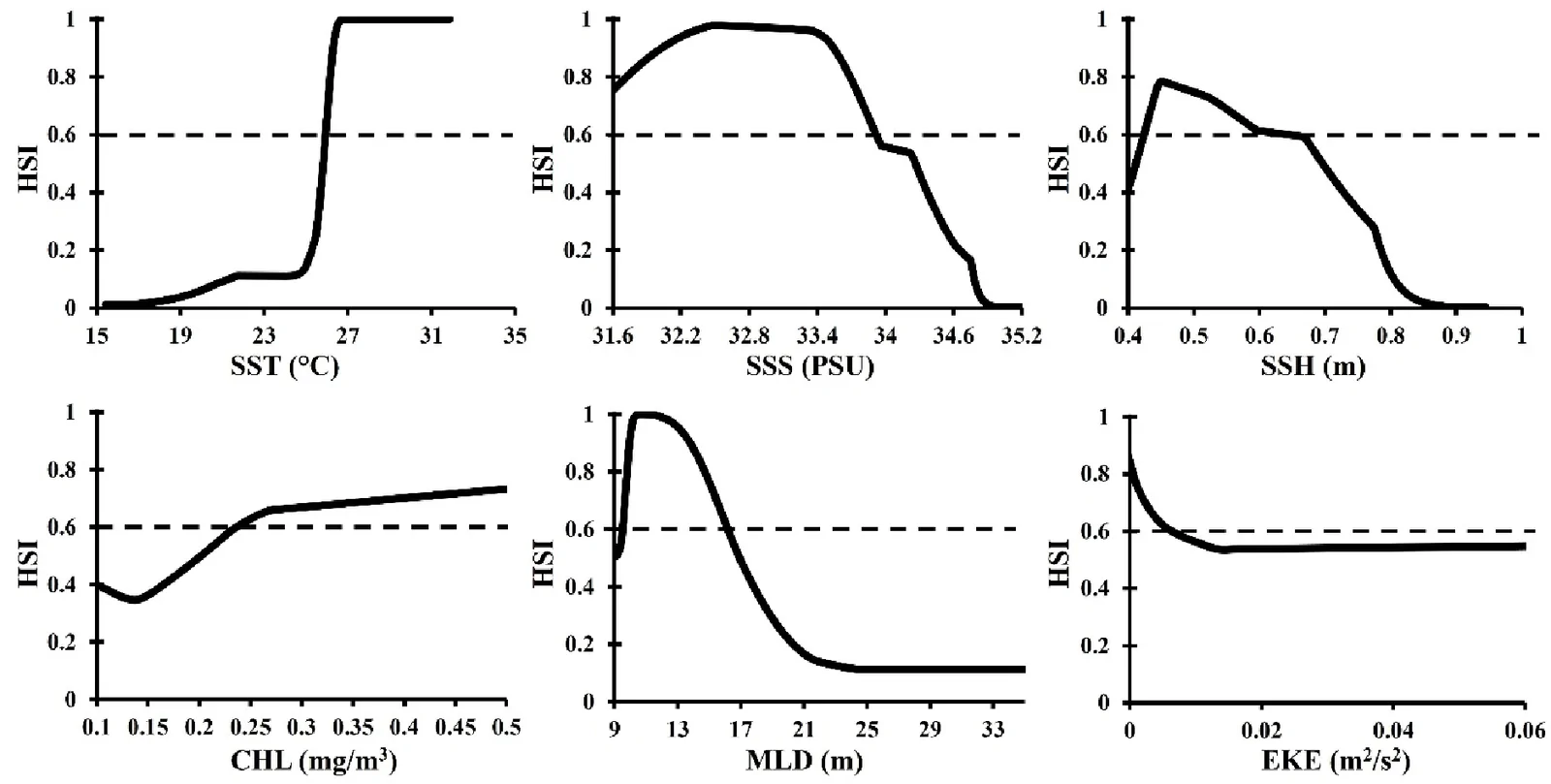
Response curves of environmental variables to habitat suitability derived from the MaxEnt model by overall. Horizontal dashed lines indicate suitable environmental variable ranges for greater amberjack.

A HSI model was developed for each oceanographic variable to characterize the specific habitat preferences of greater amberjack. Optimal environmental ranges, derived from these SI curves, were used to delineate preferred conditions based on habitat suitability scores. Seasonally, optimal SST ranges were identified as > 25 °C in spring, > 26 °C in summer, > 25 °C in autumn, and 21–26 °C in winter (Fig. 5). For SSS, optimal conditions were 32.2–34.7 PSU during spring and winter, and 31–34 PSU in summer and autumn (Fig. 5). Optimal SSH ranges were similar in spring (0.41–0.68 m) and summer (0.43–0.59 m), while autumn and winter showed ranges of 0.54–0.78 m and 0.53–0.74 m, respectively (Fig. 5). CHL were optimally lower in summer (0–0.1 mg/m^3^), moderate in spring and autumn (0.05–0.22 mg/m^3^), and highest in winter (0.2–0.5 mg/m^3^) (Fig. 5). Furthermore, the optimal MLD range was lower in spring and summer (7–17.5 m) and higher in autumn (7–20 m) and winter (> 30 m) (Fig. 5). EKE displayed a consistent optimal range of 0.011–0.019 m^2^/s^2^ across all seasons (Fig. 5). Overall, environmental conditions associated with high HSI values for greater amberjack included SST > 24 °C, SSS < 35 PSU, SSH between 0.41 and 0.64 m, CHL > 0.22 mg/m^3^, MLD between 10 and 17 m, and EKE between < 0.007 m^2^/s^2^ (Fig. 6).

## Discussion

### Habitat suitability and environmental preferences

The observed distributions are consistent with prior research indicating that greater amberjack prefer specific environmental conditions, such as SSTs ranging from 22 °C to 32 °C and SSS values from 32.4 to 35.8 PSU, for optimal habitat^17^. These findings align with the understanding that species-specific biological needs, such as spawning and feeding, drive seasonal habitat selection in marine organisms^8^. For instance, fluctuations in environmental variables like SST and MLD significantly influence the migration patterns and distribution of pelagic species^7^. These environmental factors, including SST, CHL and vertical water temperature, play a significant role in shaping the habitat patterns of marine species. For instance, these parameters strongly influence the distribution of humboldt squid in equatorial waters^37^, and variations in environmental and climatic conditions similarly affect the spatiotemporal distribution of yellowfin tuna^38^. In particular, these abiotic parameters influence the availability of prey and the physiological comfort of the greater amberjack, thereby shaping their spatial distribution and foraging behaviors^12^. The coastal waters surrounding Taiwan, forming part of the South China Sea and the East China Sea, are shaped by the influence of the Coastal Current, Taiwan Warm Current, and Kuroshio Current. This dynamic region is characterized by favorable hydrological conditions, rich nutrient availability, and a profusion of food organisms^39^ creating an ideal habitat for greater amberjack. During the summer months, the South China Sea Current and the Kuroshio Branch Current predominantly influence the region, advecting warm, high-salinity, and oligotrophic waters. Conversely, the China Coastal Current, driven by robust northeasterly winds, transports cold, low-salinity, and nutrient-enriched waters southward along the western coast of Taiwan^40^. Elucidating the interrelationship between the spatial distribution of fish assemblages and marine environmental parameters facilitates the precise identification of pelagic fish habitats through remote sensing. Moreover, habitat suitability is augmented under optimal environmental conditions, observed both seasonally and overall (Figs. 5 and 6). This pronounced seasonal and overall variability are particularly instrumental in dissecting these relationships, illustrating the non-linear responses of marine species to varying environmental conditions^41^. This detailed characterization of environmental preferences, derived directly from the SI curves, strengthens our ability to predict species responses to future climate scenarios and to delineate dynamic protected areas that account for seasonal variability in habitat use.

### The maximum entropy model and its utility for species distribution

The models utilized in this investigation consistently achieved AUC values above 0.8, indicating strong discriminatory ability in distinguishing suitable from unsuitable habitats. Nevertheless, as these AUC values are derived from presence-background comparisons rather than true presence-absence data, they may be susceptible to inflation and should be interpreted accordingly. The incorporation of complementary metrics such as the continuous Boyce index in future studies would provide a more robust and background-independent assessment of model performance. The TSS values were consistently high, indicating a high degree of predictive accuracy and a reduced incidence of error in predicting the species presence. Furthermore, the consistency across various model evaluation metrics reinforces the reliability of the MaxEnt framework for assessing habitat suitability, especially for species where presence-only data are prevalent^3^. This robustness is particularly beneficial for species like the greater amberjack, for which extensive presence-absence data can be challenging to acquire^2^. These predictive capabilities are especially critical for highly migratory species, as accurately modeling their distribution and habitat preferences is essential for effective conservation and management strategies. However, the intricate interplay between species and their environmental factors, which dictates potential geographical distributions, presents significant challenges in terms of quantification and comprehensive understanding^42,43^. The MaxEnt model characterizes the spatial distribution of species through the application of the maximum entropy principle^23,25^. This framework has been widely utilized for forecasting the spatial occurrences of terrestrial species, such as insects^44^, and has seen extensive application in analyzing both current and projected distributional patterns of marine species^2,8,26^. This current investigation further exemplifies MaxEnt’s capability to assess seasonal dynamics in the distribution of marine species across varying temporal scales. It leverages environmental covariates at species presence and background points to find the probability distribution of maximum entropy, thereby enabling reliable predictions of habitat suitability^45^.

This investigation revealed that the spatial distribution of SST and oceanic fronts significantly influenced seasonal variations within the complex hydrological environment, a finding consistent with the broader oceanographic system observed in Taiwanese waters (Fig. S2 & S3). The spatial and temporal distribution of greater amberjack within this region is primarily concentrated in oceanic frontal zones. MaxEnt models further elucidated that MLD and SST were significant determinants of habitat suitability for the greater amberjack (Fig. 2). However, the substantial seasonal movement of predicted habitats (Fig. 3) necessitates a fundamental shift towards dynamic monitoring and managing the resources, recognizing that static approaches are insufficient for effectively safeguarding species resilience against evolving environmental fluctuations and future climate uncertainties^33,46^.

The habitat ranges of greater amberjack exhibited distinct seasonal variation (Fig. 3). High habitat suitability was concentrated in the northwestern waters of Taiwan during spring, expanding southwestward and into the East China Sea in summer. Which is consistent with other studies showing how marine species tend to relocate and alter their geographical range in response to changing marine conditions^10^. The distribution contracted to its widest extent in autumn and subsequently expanded again across the northwestern and northeastern waters in winter. These observed seasonal shifts underscore the plasticity of greater amberjack species in adapting their spatial distributions to dynamic environmental conditions^3,11^.

### Limitation and prospectus of this study

Despite the robust predictive capabilities of the MaxEnt models, certain limitations merit consideration when interpreting the results. For instance, while MaxEnt excels with presence-only data, the absence of verified absence data can sometimes limit the model’s ability to precisely delineate the boundaries of unsuitable habitats. Furthermore, the reliance on fishery-dependent data, even if expert-reviewed, introduces potential biases related to sampling effort and gear selectivity, which may not fully capture the entire spectrum of the species’ habitat preferences or distribution patterns across all life stages^8^. A specific limitation concerns the designation of pseudoabsence points: in this study, areas without recorded fishing effort were treated as pseudoabsence candidates. However, this approach may introduce spatial bias if fishing vessels systematically avoid environmentally suitable areas due to operational or socioeconomic constraints unrelated to species availability. To reduce this bias, future studies should consider alternative background selection strategies, such as target-group background sampling which uses the spatial distribution of fishing effort itself as the background or environmentally stratified random background selection, both of which are more robust approaches for fishery-dependent presence-only modelling. Additionally, the availability of comprehensive biological and ecological data, such as age structure, reproductive status, and trophic interactions, could provide a more holistic understanding of the environmental drivers influencing habitat selection and distribution patterns, which current models might only partially address^3^. Therefore, future research should consider these factors. Additionally, there are differences in the methodologies and resolutions of environmental datasets utilized across different studies, leading to discrepancies in predictive accuracy and generalizability^22,35,47^. Additionally, although the default MaxEnt settings were used in this study, future studies should consider formal model tuning approaches, such as ENMeval^48^, to optimize model parameters and further improve model robustness and generalizability. Therefore, integrating the stock structure of greater amberjack into population dynamics is essential for developing more meaningful resource conservation and management strategies and establishing a robust scientific basis for these endeavors.

## Supplementary Information

Below is the link to the electronic supplementary material.

Supplementary Material 1

## Data Availability

No datasets were generated or analysed during the current study.

## References

[1] Mugo, R. & Saito, S. Ensemble Modelling of Skipjack Tuna (*Katsuwonus pelamis*) Habitats in the Western North Pacific Using Satellite Remotely Sensed Data; a Comparative Analysis Using Machine-Learning Models. *Remote Sens.* **12**, 2591 (2020).

[2] Tang, W. et al. Habitat for *Coilia nasus* in southern Zhejiang Province, China, based on a maximum entropy model. *Sci Rep* **14**, 19254 (2024). 10.1038/s41598-024-70044-y PMC11336139 39164421

[3] Mammel, M. et al. Habitat changes and catch rate variability for greater amberjack in the Taiwan Strait: The effects of El Niño–southern oscillation events. *Front Mar. Sci* **10**, 1024669 (2023).

[4] Syah, A. F., Siregar, E. S. Y., Siregar, V. P. & Agus, S. B. Application of remotely sensed data and maximum entropy model in detecting potential fishing zones of Yellowfin tuna (*Thunnus albacares*) in the eastern Indian Ocean off Sumatera. *J. Phys. Conf. Ser.* **1569**, 42097 (2020).

[5] Syah, A. F., Ni’am, A. K. & Jatisworo, D. Potential fishing grounds of Skipjack tuna (*Katsuwonus pelamis*) in western water of Sumatera using remotely sensed data and maximum entropy model. in IOP *Conference Series Earth and Environmental Science ***1251**, 12066 (IOP Publishing, 2023).

[6] Syah, A. F., Saito, S., Alabia, I. D. & Hirawake, T. Predicting potential fishing zones for Pacific saury (*Cololabis saira*) with maximum entropy models and remotely sensed data. *Fish. Bull.* **114**, 330 (2016).

[7] Liu, H. & Wei, Y. Modeling the potential distribution of Argentine shortfin squid in the southwest Atlantic Ocean. *J. Oceanol. Limnol.* 10.1007/s00343-024-4048-2 (2024).

[8] Teng, S. Y. et al. Modeling the Habitat Distribution of *Acanthopagrus schlegelii* in the Coastal Waters of the Eastern Taiwan Strait Using MAXENT with Fishery and Remote Sensing Data. *J. Mar. Sci. Eng.* **9**, 1442 (2021).

[9] Bang, M. et al. Future changes in the seasonal habitat suitability for anchovy (*Engraulis japonicus*) in Korean waters projected by a maximum entropy model. *Front Mar. Sci* **9**, 922020 (2022).

[10] Wang, J. & Tabeta, S. MaxEnt modeling to show patterns of coastal habitats of reef-associated fish in the South and East China Seas. *Front Ecol. Evol* **11**, 1027614 (2023).

[11] Mammel, M. et al. Variability in the Spatiotemporal Distribution Patterns of Greater Amberjack in Response to Environmental Factors in the Taiwan Strait Using Remote Sensing Data. *Remote Sens.* **14**, 2932 (2022).

[12] Mammel, M. et al. Unraveling the feeding strategies of the greater amberjack: insights into size–dependent dietary patterns and environmental influences in Taiwanese waters. *Fish. Sci.* 10.1007/s12562-024-01808-8 (2024).

[13] Musimwa, R. et al. Climate-induced habitat suitability modelling for pelagic fish in European seas. *Front Mar. Sci* **12**, 1501751 (2025).

[14] Su, Y., Su, S. H., Li, H. Y., Wang, H. & Lee, S. C. Implication of single year seasonal sampling to genetic diversity fluctuation that coordinates with oceanographic dynamics in torpedo scads near Taiwan. *Sci Rep* **10**, 16829 (2020). 10.1038/s41598-020-74025-9 PMC7544891 33033371

[15] Chang, Y. et al. Trophic Dynamics and Feeding Ecology of Skipjack Tuna (*Katsuwonus pelamis*) off Eastern and Western Taiwan. *Molecules* **27**, 1073 (2022). 35164337 10.3390/molecules27031073 PMC8838005

[16] Hsiao, P. Y. et al. Impacts of El Niño–Southern Oscillation (ENSO) Events on Trophodynamic Structure and Function in Taiwan Bank Marine Ecosystem. *Diversity* **16**, 572 (2024).

[17] Mammel, M., Azeez, P. A., Wang, Y., Lan, Y. & Yeh, H. Trophic ecology of the greater amberjack in relation to marine environmental characteristics in Taiwanese waters illustrated by Stable Isotope and Stomach Content Approach. *Reg. Stud. Mar. Sci.* **74**, 103553 (2024).

[18] Kobari, T. et al. Phytoplankton growth and consumption by microzooplankton stimulated by turbulent nitrate flux suggest rapid trophic transfer in the oligotrophic Kuroshio. *Biogeosciences* **17**, 2441 (2020).

[19] Li, H., Chen, Y., Li, X., Zhou, P. & Xiong, X. Assessing larval fish diversity and conservation needs in the Luzon strait using DNA barcoding. *Front Mar. Sci* **10**, 1268399 (2023).

[20] Teng, S. N., Xu, C. & Svenning, J. Upslope agricultural expansion caused mammal range contractions in China over the past two millennia. *Research Portal Denmark* (2017).

[21] Belkin, I. M. & O’Reilly, J. E. An algorithm for oceanic front detection in chlorophyll and SST satellite imagery. *J. Mar. Syst.* **78**, 319 (2009).

[22] Naimi, B., Hamm, N., Groen, T. A., Skidmore, A. K. & Toxopeus, A. G. Where is positional uncertainty a problem for species distribution modelling? *Ecography* **37**, 191 (2013).

[23] Phillips, S. J., Anderson, R. P. & Schapire, R. E. Maximum entropy modeling of species geographic distributions. *Ecol. Model.* **190**, 231 (2006).

[24] O’Brien, R. M. A Caution Regarding Rules of Thumb for Variance Inflation Factors. *Qual. Quant.* **41**, 673 (2007).

[25] Phillips, S. J. & Dudík, M. Modeling of species distributions with Maxent: new extensions and a comprehensive evaluation. *Ecography* **31**, 161 (2008).

[26] Alabia, I. D. et al. Seasonal potential fishing ground prediction of neon flying squid (*Ommastrephes bartramii*) in the western and central North Pacific. *Fish. Oceanogr.* **24**, 190 (2015).

[27] Phillips, S. A. & Brief Tutorial on Maxent. 108 (2005). 10.5531/cbc.linc.3.1.6

[28] Phillips, S. J. et al. Sample selection bias and presence-only distribution models: implications for background and pseudo‐absence data. *Ecol. Appl.* **19**, 181 (2009). 19323182 10.1890/07-2153.1

[29] Naimullah, M. et al. Effect of climate change on habitat suitability and recruitment dynamics of swimming crabs in the Taiwan Strait. *Mar Freshw. Res* **75**, 24002 (2024).

[30] Hanberry, B. B., He, H. S. & Palik, B. J. Pseudoabsence Generation Strategies for Species Distribution Models. *PLoS ONE* **7**, 44486 (2012). 10.1371/journal.pone.0044486 PMC3432107 22952985

[31] Nataniel, A., Pennino, M. G., López, J. & Soto, M. Modelling the impacts of climate change on skipjack tuna (*Katsuwonus pelamis*) in the Mozambique Channel. *Fish. Oceanogr.* **31**, 149 (2021).

[32] Manel, S., Williams, H. C. & Ormerod, S. J. Evaluating presence-absence models in ecology: the need to account for prevalence. *J. Appl. Ecol.* **38**, 921 (2001).

[33] Venne, S. & Currie, D. J. Can habitat suitability estimated from MaxEnt predict colonizations and extinctions? *Divers. Distrib.* **27**, 873 (2021).

[34] Yackulic, C. B. et al. Presence-only modelling using MAXENT: when can we trust the inferences? *Methods Ecol. Evol.* **4**, 236 (2013).

[35] Allouche, O., Tsoar, A. & Kadmon, R. Assessing the accuracy of species distribution models: prevalence, kappa and the true skill statistic (TSS). *J. Appl. Ecol.* **43**, 1223 (2006).

[36] Hernández, P. A. R., Graham, C. H., Master, L. L. & Albert, D. L. The effect of sample size and species characteristics on performance of different species distribution modeling methods. *Ecography* **29**, 773 (2006).

[37] Fang, X., Yu, W., Chen, X. & Zhang, Y. Response of Abundance and Distribution of Humboldt Squid (*Dosidicus gigas*) to Short-Lived Eddies in the Eastern Equatorial Pacific Ocean From April to June 2017. *Front Mar. Sci* **8**, 721291 (2021).

[38] Wu, Y., Lan, K., Evans, K., Chang, Y. & Chan, J. Effects of decadal climate variability on spatiotemporal distribution of Indo-Pacific yellowfin tuna population. *Sci Rep* **12**, 13715 (2022). 10.1038/s41598-022-17882-w PMC9374684 35962132

[39] Huang, T., Chen, C. A., Bai, Y. & He, X. Elevated primary productivity triggered by mixing in the quasi-cul-de-sac Taiwan Strait during the NE monsoon. *Sci Rep* **10**, 7846 (2020). 10.1038/s41598-020-64580-6 PMC7217951 32398711

[40] Chen, K., Chen, C. Y., Chang, Y., Chen, H. S. & Chen, M. A 2°C difference affecting the spatiotemporal distribution of small demersal fish assemblages in shallow tropical and subtropical waters of Western Taiwan. *Sci Rep* **13**, 20113 (2023). 10.1038/s41598-023-47300-8 PMC10656438 37978224

[41] Fusi, M. et al. The predictability of fluctuating environments shapes the thermal tolerance of marine ectotherms and compensates narrow safety margins. *Sci Rep* **14**, 26174 (2024). 10.1038/s41598-024-77621-1 PMC11526141 39478107

[42] Rao, R. et al. The species distribution model based on the random forest algorithm reveals the distribution patterns of *Neophocaena asiaeorientalis*. *Sci Rep* **15**, 10037 (2025). 10.1038/s41598-025-92508-5 PMC11930967 40122919

[43] Coll, M., Pennino, M. G., Steenbeek, J., Solé, J. & Bellido-Millán, J. M. Predicting marine species distributions: Complementarity of food-web and Bayesian hierarchical modelling approaches. *Ecol. Model.* **405**, 86 (2019).

[44] Jiang, D., Chen, S., Hao, M., Fu, J. & Ding, F. Mapping the Potential Global Codling Moth (*Cydia pomonella L.*) Distribution Based on a Machine Learning Method. *Sci Rep* **8**, 13093 (2018). 10.1038/s41598-018-31478-3 PMC6117298 30166625

[45] Sharifian, S., Mortazavi, M. S. & Nozar, S. L. M. Projected habitat preferences of commercial fish under different scenarios of climate change. *Sci Rep* **14**, 10177 (2024). 10.1038/s41598-024-61008-3 PMC11068754 38702432

[46] Silva, C., Leiva, F. & Lastra, J. A. Predicting the current and future suitable habitat distributions of the anchovy (*Engraulis ringens*) using the Maxent model in the coastal areas off central-northern Chile. *Fish. Oceanogr.* **28**, 171 (2018).

[47] Dong, Bai, X., Zhao, L., Dong, H. & Liu, C. Comparative analysis of climate-induced habitat shift of economically significant species with diverse ecological preferences in the Northwest Pacific. *Front Mar. Sci* **11**, 1476097 (2024).

[48] Muscarella, R. et al. An R package for conducting spatially independent evaluations and estimating optimal model complexity for Maxent ecological niche models. *Methods Ecol. Evol.* **5**, 1198–1205 (2014).

