# Supplementary material for: Predicting the suitable habitat distribution of greater amberjack using the MaxEnt model with fishery and remote sensing data in the surrounding waters of Taiwan
Source: Sci Rep. 2026 Sep 9;16:28231. doi: 10.1038/s41598-026-60453-6 (PMC13558566; doi:10.1038/s41598-026-60453-6)
Supplement: Supplementary file 1 [file 41598_2026_60453_MOESM1_ESM.docx]

**Supplementary Information**

**Figure S1.** Spatial distribution of Taiwanese vessels fishing locations and catch rates (kg/hour) for the greater amberjack fishery throughout the seasonal map in the surrounding waters of Taiwan. Map generated using IDL (Interactive Data Language, version 8.4; https://www.nv5geospatialsoftware.com).

**Figure S2.** Seasonal sea surface temperature (SST) changes during winter, spring, summer, and autumn from 2014 to 2019 in the surrounding waters of Taiwan. Map generated using MATLAB software (version R2019a; https://www.mathworks.com).

**Figure S3.** Seasonal oceanic frontal changes during winter, spring, summer, and autumn from 2014 to 2019 in the surrounding waters of Taiwan. Map generated using MATLAB software (version R2019a; https://www.mathworks.com).

**Figure S4.** Pairwise Pearson correlation matrix among the six oceanographic environmental predictor variables.

**Figure S5.** The variance inflation factor (VIF) analysis for each environmental factors.

**Figure S6.** The jackknife analysis of all the environmental variables included in the MaxEnt model for each season for greater amberjack.

**Figure S7.** The jackknife analysis of all the environmental variables included in the MaxEnt model for overall data for greater amberjack.
